# Glycerol‐Regulated Cytocompatible, Self‐Healing Gel Electrolyte Stabilizes Zinc Metal Anodes for Quasi‐Solid‐State Aqueous Batteries

**DOI:** 10.1002/advs.78625

**Published:** 2026-11-03

**Authors:** Yuchuan Ren, Qian Xue, Lihong He, Canhuang Li, Ruixia Li, Xiaoyu Bi, Jiali Chai, Yapeng Cheng, Tiezhu Guo, Andreu Cabot

**Affiliations:** ^1^ Catalonia Energy Research Institute – IREC Barcelona Catalonia Spain; ^2^ Departament d'Enginyeria Electrònica i Biomèdica Universitat de Barcelona Barcelona Catalonia Spain; ^3^ Hospital of Stomatology Sun Yat‐sen University Guangzhou People's Republic of China; ^4^ Guangdong Provincial Key Laboratory of Stomatology Guangzhou People's Republic of China; ^5^ Guanghua School of Stomatology Sun Yat‐sen University Guangzhou People's Republic of China; ^6^ State Key Laboratory of Tropic Ocean Engineering Materials and Materials Evaluation School of Marine Technology and Equipment Hainan University Haikou People's Republic of China; ^7^ State Key Laboratory of Cancer Biology National Clinical Research Center for Digestive Diseases Xijing Hospital of Digestive Diseases Xi'an People's Republic of China; ^8^ Multifunctional Materials and Structures Key Laboratory of the Ministry of Education & International Center for Dielectric Research School of Electronic Science and Engineering Xi'an Jiaotong University Xi'an People's Republic of China; ^9^ ICREA Barcelona Catalonia Spain

**Keywords:** cytocompatibility, gel electrolyte, hydrogen‐bond regulation, self‐healing, zinc metal anode, Zn^2^^+^ solvation, Zn‐S battery

## Abstract

Aqueous zinc batteries are attractive for safe and sustainable energy storage, but their practical development is strongly limited by the instability of Zn metal in water‐containing electrolytes, which promotes corrosion, parasitic reactions, nonuniform ion transport, and dendritic deposition. Here, we develop a glycerol‐regulated cytocompatible, self‐healing gel electrolyte (SHGE) based on a poly(vinyl alcohol)/poly(ethylene glycol) matrix. Glycerol reorganizes the hydrogen‐bonding environment and reduces polymer crystallinity, thereby decreasing water reactivity while enhancing polymer‐chain mobility and ionic transport. The resulting gel also modifies the Zn^2+^ coordination environment and promotes more regulated interfacial ion transfer, suppressing Zn corrosion and enabling compact and homogeneous Zn deposition. Consequently, Zn||Zn symmetric cells sustain stable plating/stripping for over 3300 h at 5 mA cm^−^^2^ and 5 mAh cm^−^^2^. The SHGE further exhibits low in vitro cytotoxicity toward multiple cell types, together with good flexibility and self‐healing capability. As a proof of concept for quasi‐solid‐state full‐cell operation, trace thiourea is introduced to promote sulfur redox conversion in Zn–S cells, enabling reversible cycling with high Coulombic efficiency and durable pouch‐cell operation. These results establish a multifunctional gel‐electrolyte strategy for stabilizing Zn metal anodes while integrating mechanical adaptability and cytocompatibility in quasi‐solid‐state aqueous batteries.

## Graphical abstract

A glycerol‐regulated PVA/PEG self‐healing gel electrolyte reconstructs the hydrogen‐bonding and Zn^2^^+^ coordination environments, suppressing water‐related parasitic reactions and enabling regulated Zn nucleation and homogeneous deposition. The resulting quasi‐solid‐state electrolyte combines durable Zn‐metal reversibility, self‐healing functionality, and cytocompatibility, while supporting reversible Zn–S full‐cell operation.

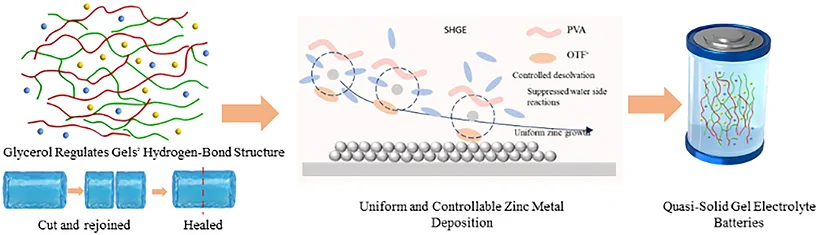

## 1 Introduction

The rapid development of wearable electronics, implantable sensors, and flexible electronic devices has created increasing demand for energy‐storage systems combining operational safety, mechanical adaptability, and compatibility with biological environments [1, 2, 3]. Aqueous zinc (Zn)‐based batteries are attractive for these applications because nonflammable aqueous electrolytes provide favorable safety characteristics, while metallic Zn offers low cost, natural abundance, and a high theoretical gravimetric capacity of 820 mAh g^−^^1^ [4, 5, 6]. Moreover, the possibility of replacing conventional liquid electrolytes with quasi‐solid‐state (QSS) configurations provides additional advantages in terms of electrolyte retention, leakage resistance, mechanical flexibility, and intimate electrode/electrolyte contact [1, 4, 7, 8]. Nevertheless, the practical implementation of such systems remains strongly constrained by the limited reversibility of Zn metal in aqueous environments.

The thermodynamic instability of Zn in water‐containing electrolytes can trigger hydrogen evolution, corrosion, surface passivation, and nonuniform Zn deposition. These interconnected parasitic processes continuously consume active Zn and electrolyte, increase interfacial resistance, and promote local current‐density inhomogeneities that may ultimately lead to dendritic growth and premature cell failure [9, 10, 11, 12]. Gel polymer electrolytes provide a promising strategy to address these challenges because they can simultaneously regulate the local electrolyte environment, maintain intimate contact with the Zn surface, and provide mechanical confinement while reducing the leakage associated with conventional liquid electrolytes [13, 14, 15]. Poly(vinyl alcohol) (PVA) is particularly attractive as a gel host because of its hydrophilicity, excellent film‐forming ability, abundant hydroxyl groups, and favorable cytocompatibility [16, 17, 18], while poly(ethylene glycol) (PEG) can interact with PVA through hydrogen bonding and improve the flexibility and wettability of the resulting polymer network. However, ion transport in conventional PVA‐based gels may be hindered by crystalline polymer domains, while residual mobile water remains available to participate in corrosion and hydrogen evolution at the Zn interface.

Regulating both the polymer structure and the local hydrogen‐bonding environment therefore represents an attractive route toward more reversible Zn metal interfaces. Glycerol, which contains three hydroxyl groups, can establish extensive hydrogen‐bonding interactions with water and polymer chains while acting as an efficient plasticizer. Its incorporation into PVA‐based gels can disrupt regular polymer‐chain packing, reduce crystallinity, increase segmental mobility, and reorganize the local hydrogen‐bond network [19, 20]. Such molecular‐level regulation can facilitate ionic transport while modifying the environment of water molecules and Zn^2^^+^ ions near the electrode/electrolyte interface. At the same time, dynamic hydrogen‐bonding interactions within glycerol‐containing polymer networks can provide mechanical flexibility and self‐healing capability, features particularly attractive for deformable and potentially bio‐integrated energy‐storage devices [19].

Beyond stabilizing the Zn anode, a practical QSS electrolyte must also remain compatible with the cathode chemistry of a complete battery. Sulfur (S) provides a useful model conversion‐type cathode because of its high theoretical capacity of approximately 1675 mAh g^−^^1^, low cost, and widespread availability [6, 7]. However, aqueous Zn─S chemistry is intrinsically challenged by the direct solid–solid conversion between electrically insulating S and ZnS. The poor electronic conductivity of both phases, limited reaction interfaces, and difficult electrochemical oxidation of ZnS result in sluggish kinetics, substantial voltage polarization, incomplete active‐material utilization, and limited cycling reversibility [13, 14, 15]. Redox‐active electrolyte additives offer one route to facilitate this conversion; in particular, thiourea has been reported to undergo reversible redox transformations and promote ZnS oxidation, thereby improving the reversibility of sulfur conversion [21]. Thus, Zn─S cells provide a demanding full‐cell platform with which to evaluate whether a Zn‐stabilizing gel electrolyte can also support reversible cathode chemistry.

Herein, we develop a glycerol‐regulated cytocompatible, self‐healing gel electrolyte (SHGE) based on a PVA/PEG polymer matrix for stabilizing Zn metal anodes in QSS aqueous batteries. Dynamic hydrogen‐bonding interactions among PVA, PEG, glycerol, and water provide mechanical flexibility and self‐healing capability while reorganizing the local electrolyte environment. Glycerol decreases polymer crystallinity and enhances segmental chain motion, resulting in increased ionic conductivity, while simultaneously modifying the Zn^2^^+^ coordination environment and suppressing water‐related parasitic reactions. Consequently, the SHGE mitigates Zn corrosion, regulates Zn nucleation and interfacial ion transport, and promotes compact and homogeneous Zn deposition. The gel further exhibits low in vitro cytotoxicity toward multiple cell types. As a proof of concept for full‐cell operation, trace thiourea is incorporated to promote S/ZnS conversion, enabling reversible QSS Zn─S batteries (QSSZSB) and mechanically robust pouch‐cell operation. This design establishes a multifunctional gel‐electrolyte strategy centered on highly reversible Zn metal anodes while retaining the mechanical adaptability, cytocompatibility, and full‐cell compatibility required for QSS aqueous batteries.

## 2 Results and Discussion

The design concept of the SHGE is illustrated in Figure 1a. A dynamic supramolecular network is constructed by incorporating glycerol into the PVA/PEG gel matrix. The abundant hydroxyl groups of PVA, PEG, and glycerol establish extensive intermolecular hydrogen‐bonding interactions throughout the network. These reversible interactions act as physical crosslinking sites that maintain the structural integrity of the gel while also serving as dynamic junctions capable of continuous dissociation and reformation. Upon mechanical damage, polymer chains at the fractured interfaces can re‐establish hydrogen‐bonding interactions when the separated surfaces are brought back into contact, thereby restoring the continuity of the gel network. As shown in Figure S1, the SHGE can be readily wrapped around a straight tube without visible fracture, demonstrating good flexibility. After being cut and brought into contact, the separated gel segments recover their macroscopic structural integrity within 4 h, consistent with the reformation of dynamic hydrogen bonds across the damaged interface.

**FIGURE 1:**
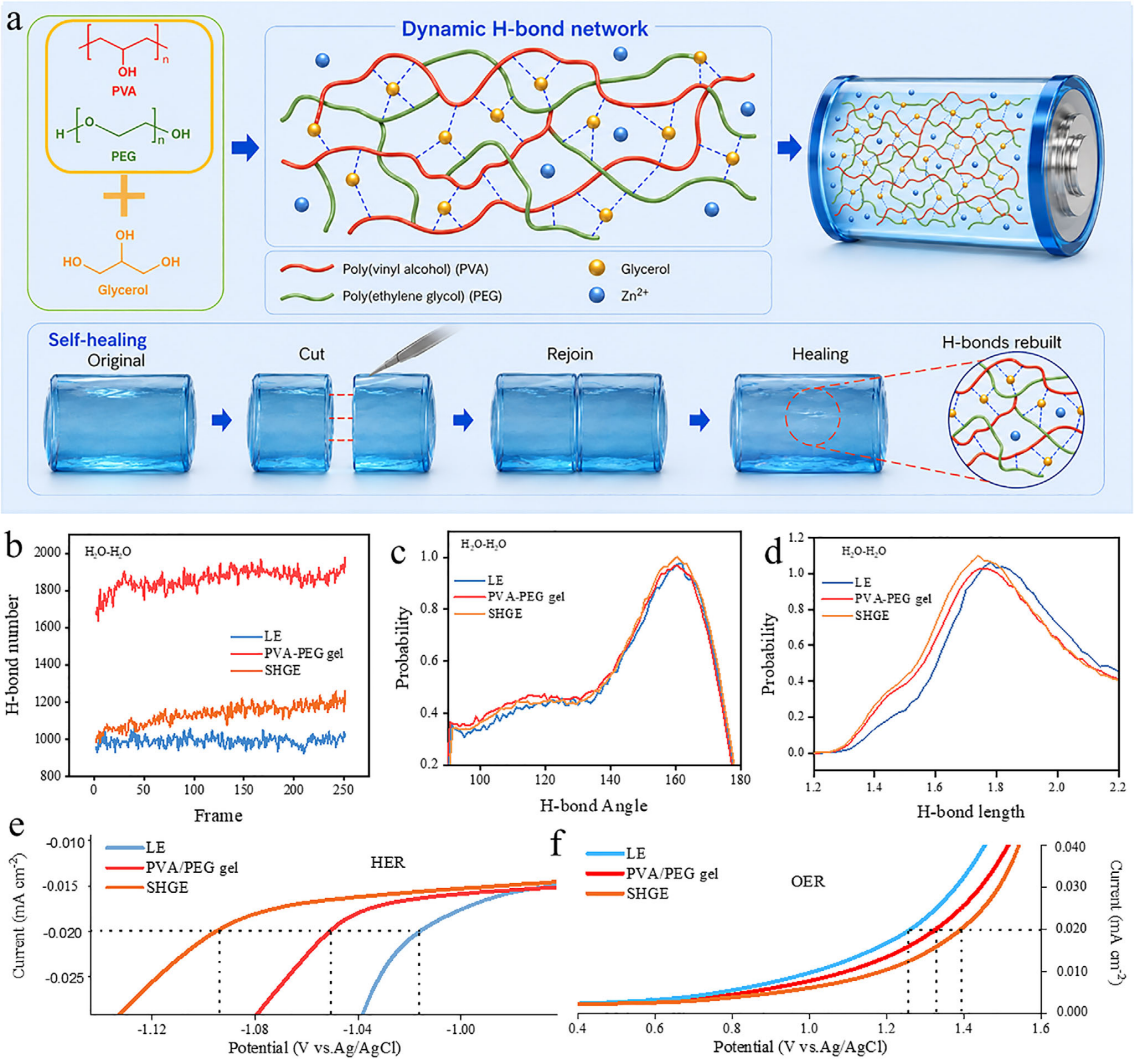
(a) Schematic illustration of the SHGE based on a PVA/PEG/glycerol network. (b–d) Molecular dynamics simulation results comparing water–water hydrogen‐bond characteristics in the LE, PVA/PEG gel electrolyte, and SHGE: b) hydrogen‐bond number, (c) hydrogen‐bond angle distribution, and (d) hydrogen‐bond length distribution. (e, f) LSV curves showing the HER and OER behavior of LE, PVA/PEG gel electrolyte, and SHGE.

To elucidate the influence of glycerol on the local hydrogen‐bonding environment, molecular dynamics simulations were performed to analyze water–water hydrogen‐bond characteristics in the different electrolyte systems. As shown in Figure 1b, the liquid electrolyte (LE) exhibits the lowest number of water–water hydrogen bonds among the three systems. Upon introducing the PVA/PEG framework, the number of water–water hydrogen bonds increases markedly, indicating that confinement within the polymer matrix alters the organization of water molecules. After glycerol incorporation, the number of water–water hydrogen bonds decreases relative to that in the PVA/PEG gel electrolyte. This reduction is attributed to the multiple hydroxyl groups of glycerol, which can establish competitive interactions with both water molecules and polymer chains, partially replacing water–water interactions and reorganizing the local hydrogen‐bond network.

Further information on the hydrogen‐bond structure was obtained from the corresponding angle and length distributions. As shown in Figure 1c, all electrolytes exhibit a dominant hydrogen‐bond angle centered at approximately 160°, suggesting that the overall hydrogen‐bond directionality remains largely unchanged after gelation and glycerol incorporation. In contrast, a clear shift is observed in the hydrogen‐bond length distribution (Figure 1d). Compared with the liquid electrolyte, both gel electrolytes display shorter water–water hydrogen‐bond distances, with SHGE showing the shortest average distance among the investigated systems. Together with the changes in hydrogen‐bond number, these results indicate that glycerol does not simply weaken or strengthen the original water network, but rather induces a substantial redistribution and reorganization of hydrogen‐bonding interactions within the gel.

Linear sweep voltammetry (LSV) was performed to evaluate the electrochemical stability windows of LE, the PVA/PEG gel electrolyte, and SHGE. As shown in Figure 1e, both gel electrolytes exhibit more negative hydrogen evolution reaction (HER) onset potentials than the liquid electrolyte, indicating suppressed hydrogen evolution. Notably, SHGE shows the most negative HER onset potential among the investigated systems. Similarly, the anodic polarization curves in Figure 1f reveal that SHGE exhibits the highest oxygen evolution reaction (OER) onset potential, indicating enhanced resistance toward oxidative water decomposition. The simultaneous suppression of HER and OER is consistent with reduced water reactivity in the glycerol‐containing gel environment [21, 22, 23] and results in a broader electrochemical stability window. In addition, glycerol provides favorable water‐retention properties to the SHGE, as shown in Figure S2. At room temperature and 50% relative humidity, the gel retains approximately 90% of its initial weight after 15 days, indicating limited solvent loss during prolonged exposure.

Fourier‐transform infrared (FTIR) spectroscopy was employed to investigate the intermolecular interactions within the gel electrolytes before and after glycerol incorporation. As shown in Figure S3, both electrolytes exhibit a broad O–H stretching band in the range of 3200–3500 cm^−^^1^, indicating extensive hydrogen‐bonding interactions among polymer chains and water molecules.22 Compared with the PVA/PEG gel electrolyte, the O─H stretching band of SHGE shifts slightly toward higher wavenumbers, indicating that glycerol modifies the original hydrogen‐bonding environment. This shift is consistent with a redistribution of hydrogen‐bond interactions among water molecules, glycerol, and the polymer chains. Meanwhile, the pronounced changes in the 1000–1200 cm^−^^1^ region, associated with C─O stretching vibrations, further confirm the incorporation of glycerol and its interaction with the polymer framework. The absorption band around 1640 cm^−^^1^, associated with molecular water, also exhibits subtle changes, supporting an altered local hydration environment [24]. Together with the molecular dynamics simulation results, these observations indicate that glycerol actively participates in the intermolecular interaction network and reorganizes the local hydrogen‐bonding environment within the gel. This reorganization is consistent with the reduced water reactivity inferred from the electrochemical measurements and may contribute to the improved electrochemical stability of the SHGE.

To further elucidate the influence of glycerol on the polymer microstructure, X‐ray diffraction (XRD) measurements were conducted for the PVA/PEG gel electrolyte and SHGE. As shown in Figure S4, the PVA/PEG gel electrolyte exhibits diffraction features at 2θ = 19.5°, 22°, and 29°, which are associated with ordered domains arising from the packing of PVA and PEG chains [25, 26]. After glycerol incorporation, the intensity of these diffraction features decreases markedly, while the peak around 29° nearly disappears, indicating a substantial reduction in polymer‐chain ordering and crystallinity. This behavior can be attributed to the insertion of glycerol molecules between adjacent polymer chains, which disrupts regular chain packing and weakens intermolecular organization within the polymer network. The resulting increase in amorphous character is generally favorable for ion transport in gel polymer electrolytes, because enhanced segmental motion in disordered regions can provide dynamic coordination environments and facilitate ion migration [17].

Consistent with this structural evolution, the ionic conductivity increases from 11.2 mS cm^−^^1^ for the PVA/PEG gel electrolyte to 28.7 mS cm^−^^1^ for SHGE (Figure S5), corresponding to an enhancement of approximately 156%. The improved ionic conductivity is consistent with the reduced crystallinity and enhanced polymer‐chain mobility induced by glycerol. These changes facilitate ion transport through the gel matrix by increasing the dynamic character of the polymer network and providing more accessible migration pathways. Therefore, glycerol not only reorganizes the hydrogen‐bonding environment but also modifies the polymer‐chain structure, jointly contributing to improved ionic transport in SHGE. To quantitatively evaluate the functional recovery of SHGE after mechanical damage, the ionic conductivity was measured before and after repeated cutting/healing processes (Figure S6). The pristine SHGE exhibits a conductivity of 28.74 mS cm^−^^1^, which recovers to 26.52 mS cm^−^^1^ after the first healing cycle, corresponding to 92.28% of the initial value. Even after a second cutting/healing process, a conductivity of 25.27 mS cm^−^^1^ is retained, corresponding to a recovery efficiency of 87.93%. The high conductivity retention after repeated damage demonstrates that the dynamic gel network can effectively reconstruct continuous ion‐transport pathways, thereby preserving the electrolyte functionality after self‐healing.

To gain molecular‐level insight into how SHGE regulates Zn^2^^+^ solvation, molecular dynamics simulations were used to analyze the local coordination environment of Zn^2^^+^ in the different electrolyte systems. As shown in Figure 2a, the radial distribution function (RDF) of Zn─O (H_2_O) in LE exhibits a prominent peak at approximately 2.0 Å, corresponding to the first solvation shell of Zn^2^^+^. The corresponding coordination number of H_2_O around Zn^2^^+^ is 5.61, indicating a predominantly hydrated Zn^2^^+^ environment. The simulated electrolyte configuration and representative solvation structure further visualize this water‐rich coordination shell, in which Zn^2^^+^ is coordinated mainly by H_2_O molecules, with more limited participation of trifluoromethanesulfonate (OTF^−^) anions (Figure 2b,c). Such a strongly hydrated environment can favor water‐related parasitic reactions and impose a substantial desolvation requirement during Zn deposition. After introducing the PVA/PEG polymer matrix, the Zn–O(H_2_O) coordination number decreases to 4.66 (Figure S7), indicating that the polymer environment modifies the water‐dominated Zn^2^^+^ solvation structure. Nevertheless, the relatively high H_2_O coordination number shows that Zn^2^^+^ remains predominantly hydrated in the PVA/PEG gel electrolyte.

**FIGURE 2:**
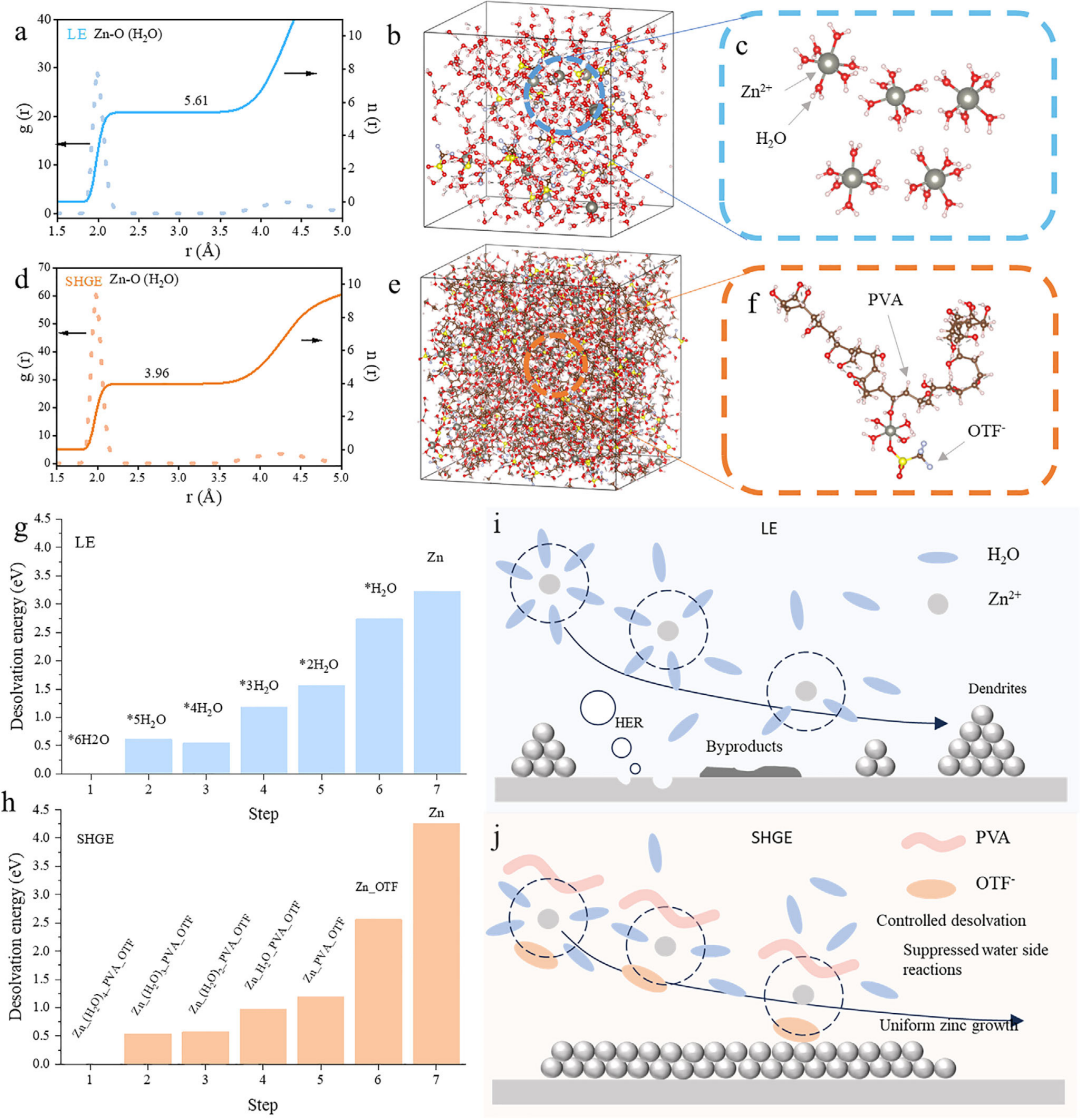
(a) Radial distribution function and coordination number of Zn─O(H_2_O) in LE. (b) Simulated molecular configuration of LE. (c) Representative Zn^2^^+^ solvation structure in LE. (d) Radial distribution function and coordination number of Zn–O(H_2_O) in SHGE. (e) Simulated molecular configuration of SHGE. (f) Representative Zn^2^^+^ solvation structure in SHGE. (g, h) Calculated Zn^2^^+^ desolvation‐energy profiles in (g) LE and (h) SHGE. (i, j) Schematic illustrations of Zn deposition behavior in (i) LE and (j) SHGE.

Upon glycerol incorporation, the Zn^2^^+^ coordination environment is further modified. As shown in Figure 2d, the Zn─O(H_2_O) coordination number in SHGE decreases to 3.96, lower than that in both LE and the PVA/PEG gel electrolyte. The corresponding simulated configuration and representative solvation structure (Figure 2e,f) reveal a mixed coordination environment involving H_2_O, OTF^−^, polymer segments, and glycerol. To clarify the contribution of the different coordinating species, additional RDF analyses were performed. In the PVA/PEG gel electrolyte, the coordination numbers of OTF^−^, PVA, and PEG around Zn^2^^+^ are 1.01, 0.23, and 0.07, respectively (Figure S8), indicating relatively limited direct participation of the polymer chains in Zn^2^^+^ coordination. In SHGE, the corresponding coordination numbers of OTF^−^, PVA, PEG, and glycerol are 0.81, 0.85, 0.13, and 0.22, respectively (Figure S9). Notably, the PVA contribution increases markedly from 0.23 to 0.85 after glycerol incorporation, whereas the direct coordination number of glycerol remains comparatively low. These results suggest that glycerol is not a dominant ligand in the first Zn^2^^+^ coordination shell. Instead, glycerol indirectly regulates the Zn^2^^+^ coordination environment by reorganizing intermolecular hydrogen bonding and increasing polymer‐chain flexibility, thereby facilitating greater participation of PVA segments in Zn^2^^+^ coordination. Overall, SHGE shifts the Zn^2^^+^ solvation environment from predominantly water‐rich coordination toward a more polymer‐assisted mixed coordination structure.

The calculated desolvation‐energy evolution was further examined to assess how this modified coordination environment may affect interfacial Zn^2^^+^ transfer. In LE, Zn^2^^+^ is predominantly surrounded by water molecules, and the modeled desolvation process therefore involves the stepwise removal of coordinated H_2_O molecules from the hydrated Zn^2^^+^ solvation shell (Figure 2g and Figure S10). The energetic cost increases progressively as coordinated water molecules are removed, indicating that strong Zn^2^^+^–H_2_O interactions impose a substantial energetic penalty on desolvation before Zn deposition. Such a water‐dominated coordination environment may hinder interfacial Zn^2^^+^ transfer while maintaining a high population of water molecules in the vicinity of the Zn surface, favoring hydrogen evolution, corrosion, and nonuniform Zn growth.

In contrast, the modeled desolvation pathway in SHGE reflects its polymer‐assisted mixed coordination environment (Figure 2h and Figure S11). Rather than involving only the sequential removal of H_2_O molecules, desolvation proceeds through the dissociation of H_2_O‐, PVA‐, and OTF^−^‐associated coordination units. This behavior is consistent with the reconstructed Zn^2^^+^ coordination environment induced by the glycerol‐regulated gel network. The reduced number of coordinated water molecules suggests a lower contribution of water to the Zn^2^^+^ solvation shell, potentially limiting water‐related parasitic reactions at the Zn interface. The calculated ligand‐removal energies illustrate the energetic differences between the coordination environments of Zn^2^^+^ in LE and SHGE. Although these calculations do not directly represent the free‐energy barriers for interfacial Zn^2^^+^ desolvation, they provide insight into the energetic consequences of replacing coordinated water molecules with polymer‐ and anion‐associated species. Overall, the results support the proposed redistribution of Zn^2^^+^ coordination in the glycerol‐containing gel, consistent with the experimentally observed improvements in Zn deposition behavior.

The modified Zn^2^^+^ coordination and desolvation behavior therefore provide a molecular‐level basis for stabilizing the Zn metal interface. Reduced water coordination around Zn^2^^+^ limits the amount of coordinated water associated with Zn^2^^+^ transport, while polymer‐assisted coordination can contribute to a more regulated Zn^2^^+^ distribution within the gel matrix. As schematically illustrated in Figure 2i,j, LE is characterized by a water‐rich Zn^2^^+^ solvation environment, extensive water‐related side reactions, by‐product formation, and nonuniform Zn growth. By comparison, SHGE provides a less water‐dominated coordination environment, suppresses water‐induced parasitic reactions, and promotes a more homogeneous interfacial Zn^2^^+^ flux. These effects are consistent with the improved Zn nucleation and deposition behavior observed experimentally.

The corrosion behavior of Zn metal in the different electrolytes was first evaluated by immersing Zn foils for 14 days. As shown by scanning electron microscopy (SEM) images in Figure 3a,b, the Zn foil immersed in LE exhibits a rough surface covered with abundant flake‐like corrosion products, indicating severe parasitic reactions between Zn metal and the electrolyte. In sharp contrast, the Zn foil stored in SHGE maintains a relatively smooth and compact surface without obvious accumulation of corrosion by‐products, demonstrating substantially improved Zn interfacial stability. The corresponding XRD patterns further support this observation (Figure 3c). Compared with fresh Zn, additional diffraction signals appear for the Zn foil immersed in LE, suggesting the formation of corrosion‐related by‐products, Zn_x_OTF_y_(OH)_2x−y_·*n*H_2_O, during prolonged exposure [27]. In contrast, the Zn foil stored in SHGE exhibits an XRD pattern similar to that of fresh Zn, consistent with the suppressed water reactivity and reduced formation of corrosion by‐products in the gel electrolyte.

**FIGURE 3:**
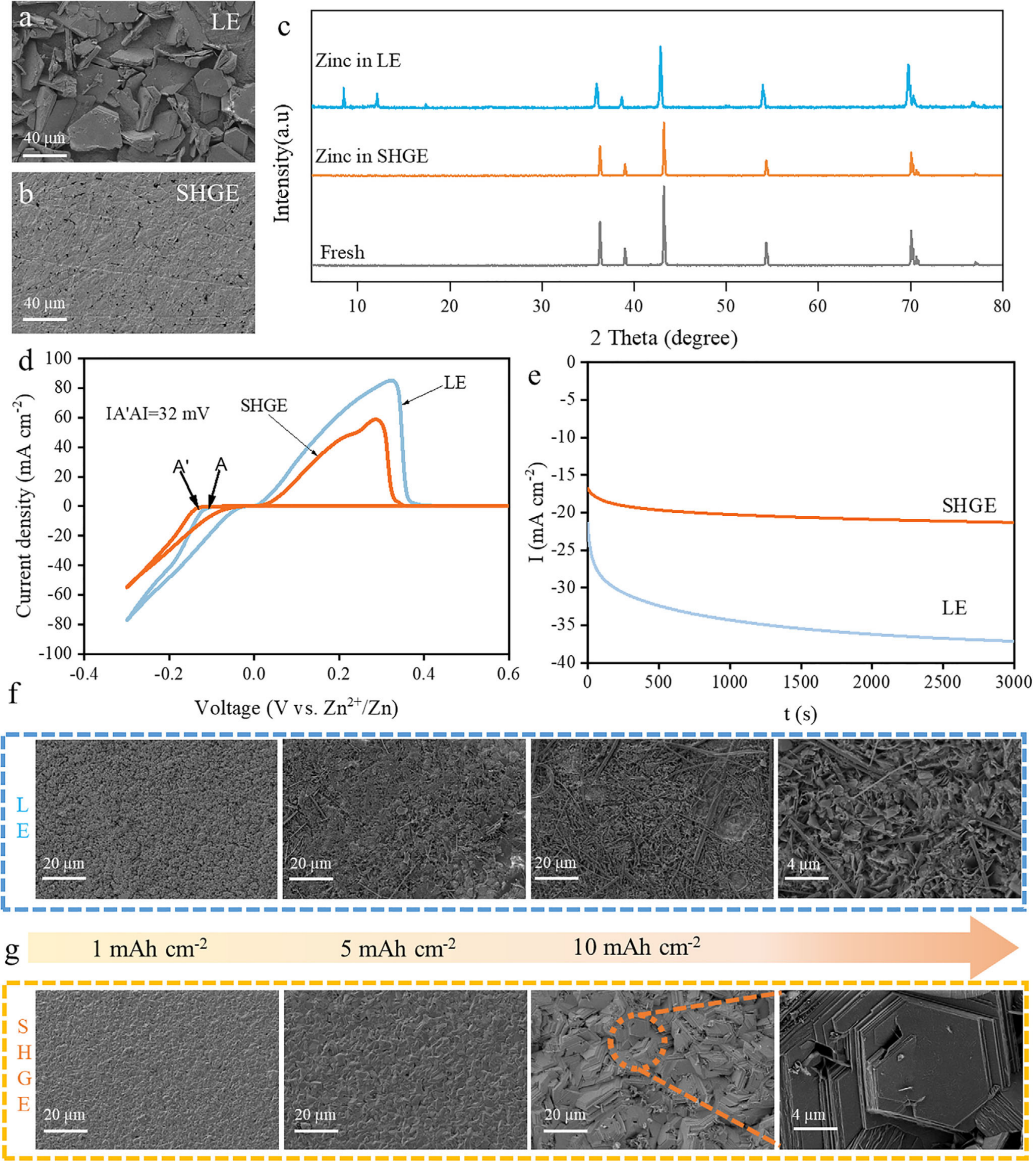
(a, b) SEM images of Zn foils after immersion for 14 days in (a) LE and (b) SHGE. (c) XRD patterns of fresh Zn and Zn foils after immersion in LE and SHGE. (d) CV curves of Zn//Cu cells in LE and SHGE. (e) Chronoamperometry curves of Zn//Zn symmetric cells at a fixed overpotential of −150 mV. (f, g) SEM images of Zn deposited on Cu substrates at different areal capacities in (f) LE and (g) SHGE.

Zn nucleation behavior was further investigated by cyclic voltammetry (CV) using Zn//Cu cells. As shown in Figure 3d, the Zn nucleation overpotential in SHGE is 32 mV higher than that in LE, indicating a higher energetic requirement for the initial nucleation of Zn on Cu. Consistently, the galvanostatic nucleation measurements in Figure S12a reveal a nucleation‐growth overpotential of 55.4 mV for SHGE, which is 29.6 mV higher than that of LE. Although a higher nucleation overpotential does not necessarily imply more uniform Zn deposition, it indicates a modified nucleation process that may influence the density and subsequent growth of Zn nuclei. At an early deposition capacity of 0.5 mAh cm^−^^2^ (Figure S12b,c), Zn deposited in SHGE exhibits finer, more uniformly distributed, and more compact deposits, whereas LE produces larger and irregular flake‐like structures. These morphological observations support the beneficial effect of SHGE on early‐stage Zn deposition, consistent with changes in Zn^2^^+^ coordination and the electrode/electrolyte interfacial environment.

To further investigate Zn deposition behavior, chronoamperometry measurements were performed in Zn//Zn symmetric cells at a fixed overpotential of −150 mV. As shown in Figure 3e, the magnitude of the current density in LE continuously increases with time, suggesting progressive changes in the electrochemically active deposition area and interfacial deposition behavior. In contrast, the current response in SHGE rapidly approaches a quasi‐steady state at a substantially lower current density, indicating distinct Zn deposition kinetics and a more stable interfacial response. Together with the SEM observations, these results are consistent with more controlled Zn growth in SHGE. It is worth noting that classical nucleation–growth models, such as the Scharifker–Hills model, rely on idealized assumptions that may not adequately describe electrodeposition in battery systems, where evolving electrode morphology, interfacial kinetics, and reactant availability can strongly influence the deposition process. As recently discussed by Yu et al., conventional nucleation models may fail to capture important features of electrodeposition under reactant‐limited conditions [28]. Considering the additional complexity introduced by the polymer network in SHGE, the chronoamperometric results are therefore interpreted qualitatively and complemented by galvanostatic nucleation measurements and direct morphological observations.

Quantitative Coulombic‐efficiency measurements are essential for evaluating the stability of Zn metal anodes [29, 30]. To quantitatively evaluate the reversibility of Zn plating/stripping, Zn||Cu asymmetric cells were further investigated. As shown in Figure S13, the SHGE‐based cell rapidly reaches a high CE after the initial activation process and maintains stable Zn plating/stripping over 1000 cycles, delivering an average CE of 99.81%. After interfacial stabilization, the average CE increases to 99.86% over cycles 100–1000 and further reaches 99.91% over cycles 500–1000, with only minor fluctuations. In contrast, the LE‐based cell exhibits substantially larger CE fluctuations during the investigated cycling period, indicating less stable Zn plating/stripping behavior. These results demonstrate that the regulated electrolyte environment in SHGE effectively suppresses irreversible Zn loss and parasitic interfacial reactions, thereby enabling highly reversible Zn plating/stripping.

The evolution of Zn deposition morphology on Cu substrates at different areal capacities and a fixed current density of 5 mA cm^−^^2^ further reveals the distinct growth behavior in the two electrolytes. As shown in Figure 3f, Zn deposited in LE already exhibits a loose and porous morphology at an areal capacity of only 1 mAh cm^−^^2^. At 5 mAh cm^−^^2^, abundant irregular Zn deposits develop on the Cu surface, together with substantial flake‐like by‐products, while Zn growth extends into the glass‐fiber separator. At 10 mAh cm^−^^2^, severe nonuniform deposition results in glass fibers becoming embedded within the loosely packed Zn deposits, evidencing uncontrolled growth under liquid‐electrolyte conditions. By contrast, Zn deposited in SHGE forms larger, compact plate‐like grains that assemble into a dense and relatively homogeneous deposit over the investigated range of areal capacities (Figure 3 g). Even at 10 mAh cm^−^^2^, the deposited Zn retains a compact morphology rather than evolving into the highly irregular and porous structures observed in LE. These results demonstrate that SHGE suppresses Zn corrosion and promotes more compact and homogeneous Zn growth, consistent with the reduced water reactivity, improved interfacial stability, and more regulated Zn^2^^+^ transport provided by the glycerol‐modified gel environment.

The long‐term reversibility of Zn plating/stripping was further evaluated using Zn||Zn symmetric cells at a current density of 5 mA cm^−^^2^ and an areal capacity of 5 mAh cm^−^^2^. As shown in Figure 4a, the cell employing LE exhibits a rapid increase in voltage polarization and undergoes an internal short circuit during the initial cycling period. In sharp contrast, the SHGE‐based symmetric cell maintains stable Zn plating/stripping for more than 3300 h with a small and nearly constant voltage hysteresis. The enlarged voltage profiles in Figure 4b show that the polarization of the LE‐based cell progressively increases after approximately 25 h, followed by pronounced voltage fluctuations and abrupt spikes, indicating progressive interfacial deterioration and unstable Zn deposition. By comparison, the enlarged profiles recorded for the SHGE‐based cell at approximately 500 and 3030 h show well‐defined and highly reproducible voltage plateaus, indicating that a stable Zn/electrolyte interface is maintained even after prolonged cycling at high areal capacity. To clarify the specific role of glycerol, a PVA/PEG gel electrolyte without glycerol was introduced as a control. As shown in Figure S14, the PVA/PEG gel exhibits better cycling stability than the liquid electrolyte, indicating that the polymer matrix itself contributes to partial interfacial stabilization. Nevertheless, the cell with the PVA/PEG gel shows progressively enlarged polarization and fails after ∼270 h, whereas the SHGE maintains highly stable cycling for over 3300 h under identical conditions. This result demonstrates that the polymer matrix alone is insufficient to ensure long‐term reversible Zn plating/stripping, and that the incorporation of glycerol is crucial for further regulating the hydrogen‐bonding network and Zn^2^^+^ coordination environment, thereby enabling much more stable Zn deposition behavior.

**FIGURE 4:**
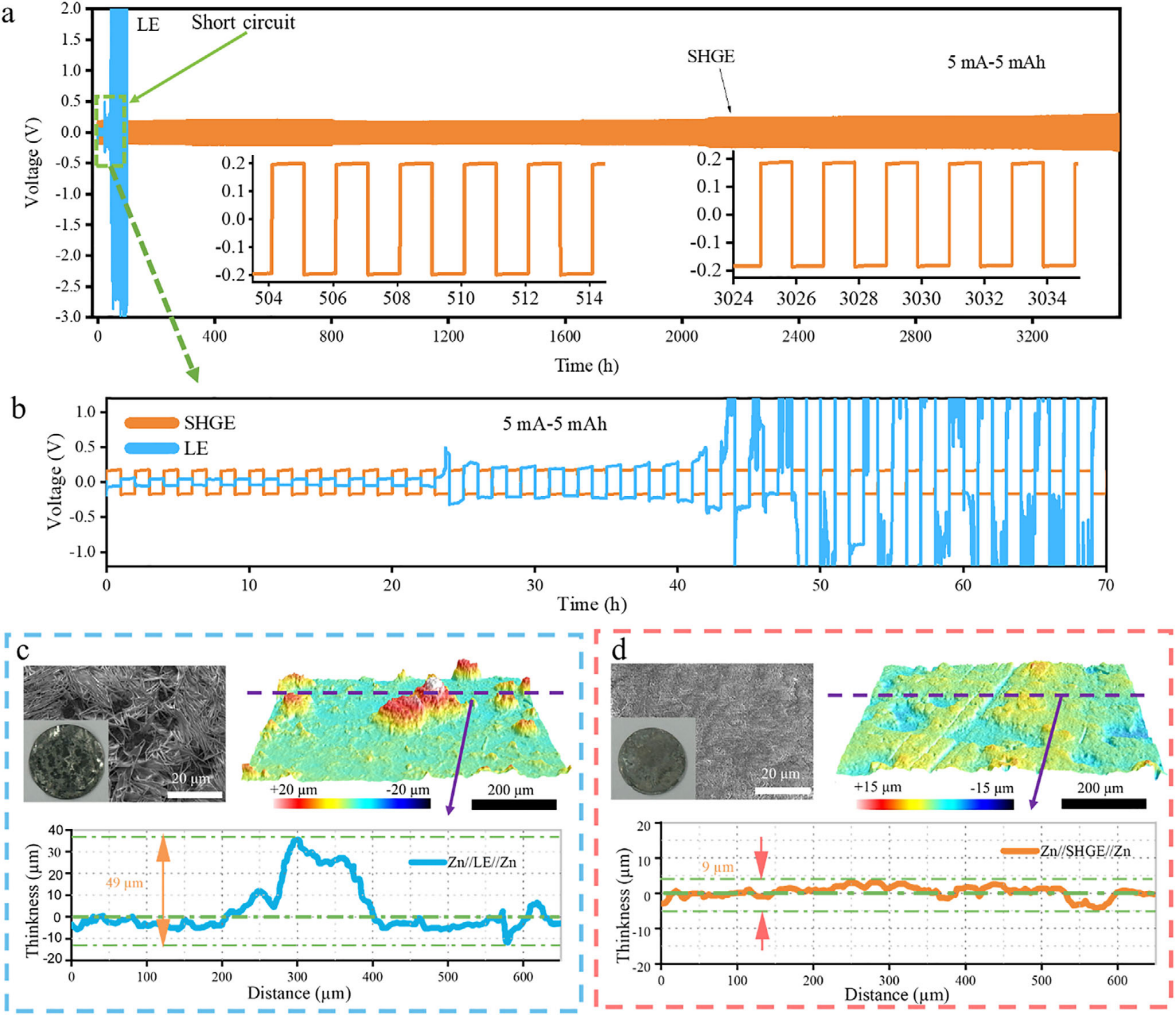
(a) Long‐term galvanostatic cycling performance of Zn||Zn symmetric cells using LE and SHGE at 5 mA cm^−^^2^ and 5 mAh cm^−^^2^. (b) Enlarged voltage profiles at selected cycling stages. (c, d) SEM images, optical photographs, three‐dimensional surface topography, and corresponding height profiles of Zn electrodes after cycling in (c) LE and (d) SHGE.

The markedly different cycling behaviors were further correlated with the post‐cycling morphology and surface‐height distribution of the Zn electrodes. After cycling in LE (Figure 4c), the Zn surface is covered with abundant needle‐like and vertically protruding deposits and exhibits a highly nonuniform surface profile. The three‐dimensional topographic image reveals pronounced local protrusions, with a maximum surface‐height variation of approximately 49 µm, consistent with severe nonuniform Zn accumulation and dendritic growth. Such heterogeneous deposition increases the local surface roughness and can intensify electric‐field and current‐density inhomogeneities, further promoting parasitic reactions and unstable Zn growth. By comparison, the Zn electrode cycled in SHGE exhibits a compact and relatively homogeneous surface without obvious dendritic protrusions (Figure 4d). Its surface‐height variation is limited to approximately 9 µm, substantially lower than that observed in LE, confirming a much more uniform Zn surface after prolonged cycling. These results demonstrate that SHGE effectively regulates Zn deposition and suppresses uncontrolled vertical growth under demanding plating/stripping conditions. The reduced water reactivity, modified Zn^2^^+^ coordination environment, and physical confinement provided by the gel collectively contribute to suppressing corrosion and dendrite formation, stabilizing the Zn/electrolyte interface and enabling prolonged reversible Zn plating/stripping.

To demonstrate the compatibility of SHGE with a complete QSS battery, sulfur was selected as a representative conversion‐type cathode. The cathode consisted of an S/C composite containing approximately 50 wt.% S (Figure S15) and guar gum mixed at a mass ratio of 9:1, with hydrophilic carbon cloth used as the current collector; further preparation details are provided in the Supporting Information (SI). Thiourea (TU) has previously been reported to undergo reversible redox transformations during Zn─S cycling, with redox‐active intermediates interacting with ZnS and facilitating its oxidation back to sulfur [21]. Therefore, TU was introduced during SHGE preparation at a concentration of 0.05 wt.% in the Zn(OTF)_2_ soaking solution for the subsequent QSSZSB tests. Unless otherwise specified, the sulfur loading was 2 mg cm^−^^2^, and all reported specific capacities and current densities were normalized to the mass of sulfur in the cathode. Figure 5a shows the CV curves of the SHGE‐based Zn–S cell during the first three cycles at a scan rate of 0.05 mV s^−^^1^. A reduction feature at approximately 1.1 V is tentatively assigned to TU‐associated redox activity based on previous reports [21], while the reduction peak around 0.15 V is attributed to the conversion of S to ZnS. The oxidation feature around 1.4 V is associated with the reverse conversion toward elemental sulfur. The substantial overlap of the CV profiles over the first three cycles indicates reproducible electrochemical behavior during the initial cycling.

**FIGURE 5:**
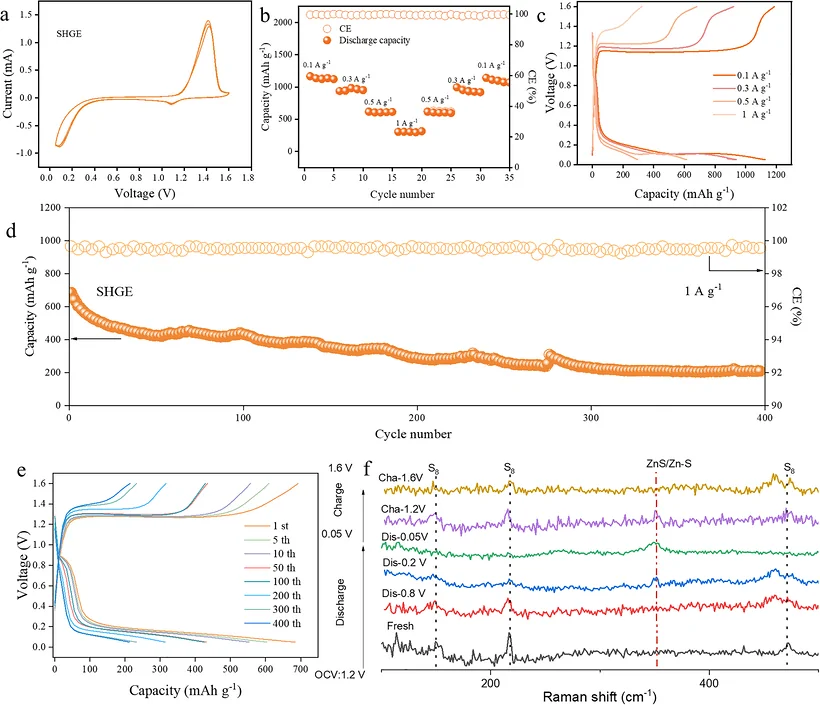
(a) CV curves of the SHGE‐based Zn─S cell during the first three cycles. (b) Rate performance at current densities from 0.1 to 1 A g^−^^1^ and corresponding Coulombic efficiency. (c) GCD profiles at different current densities. (d) Long‐term cycling performance at 1 A g^−^^1^. (e) GCD profiles at selected cycles. (f) In situ Raman spectra collected during the initial discharge and subsequent charge processes.

The rate capability was subsequently evaluated at current densities ranging from 0.1 to 1 A g^−^^1^. As shown in Figure 5b, the SHGE‐based QSSZSB delivers specific discharge capacities of 1165, 938, 615, and 304 mAh g^−^^1^ at 0.1, 0.3, 0.5, and 1 A g^−^^1^, respectively. When the current density is returned to 0.1 A g^−^^1^, a discharge capacity of 1141 mAh g^−^^1^ is recovered, while the Coulombic efficiency remains above 99.2%. The corresponding galvanostatic charge/discharge (GCD) profiles are shown in Figure 5c. In addition to the low‐voltage S‐to‐ZnS conversion plateau, a higher‐voltage discharge contribution of approximately 80 mAh g^−^^1^ is observed, which is tentatively associated with TU‐related redox activity based on previous reports. Because the specific capacity is normalized to the mass of sulfur, the reported values may include contributions from both sulfur conversion and TU‐associated redox processes. Increasing the current density leads to increased polarization, consistent with the intrinsically sluggish solid–solid conversion between S and ZnS. Nevertheless, the characteristic discharge features remain observable over the investigated current‐density range, indicating that both the TU‐mediated redox process and sulfur conversion remain electrochemically active.

Cycling stability was further evaluated at 1 A g^−^^1^. As shown in Figure 5d, the cell maintains a Coulombic efficiency close to 100% over 400 cycles. Although the discharge capacity gradually decreases during prolonged cycling, the cell continues to operate without abrupt failure, demonstrating that SHGE can support sustained full‐cell operation with a conversion‐type sulfur cathode. The charge/discharge profiles recorded at selected cycles (Figure 5e) retain the characteristic electrochemical features associated with both TU redox activity and S/ZnS conversion, further indicating that the cathode chemistry remains active during extended cycling. To distinguish the contribution of SHGE from that of the thiourea redox mediator, a Zn–S cell employing the glycerol‐free PVA/PEG gel containing the same TU concentration (0.05 *w*t.%) was evaluated as a control (Figure S16). Despite the presence of TU, the PVA/PEG‐based cell exhibits rapid capacity decay and pronounced cycling fluctuations, whereas the SHGE‐based cell maintains markedly improved capacity retention and long‐term cycling stability. Because the TU concentration is identical in both systems, this comparison confirms that TU alone is insufficient to sustain durable Zn─S cycling. Instead, SHGE provides a stable quasi‐solid‐state electrochemical environment by regulating the water and Zn^2^^+^ coordination environment, suppressing Zn‐side parasitic reactions, and maintaining reversible Zn plating/stripping, while TU mainly facilitates sulfur redox conversion.

In situ Raman spectroscopy was employed to further probe the sulfur conversion process during discharge and charge. For these measurements, the sulfur loading was increased to 15 mg cm^−^^2^ to enhance the Raman signal. As shown in Figure 5f, the pristine electrode exhibits characteristic Raman bands of S_8_ at 150.2, 217.8, and 472.0 cm^−^^1^, confirming the presence of elemental sulfur in the initial state. During discharge, the intensity of the S_8_‐related bands progressively decreases, accompanied by the appearance of a vibration band at approximately 352.0 cm^−^^1^, tentatively assigned to ZnS/Zn–S‐related species. Upon subsequent charging, this band decreases while the characteristic S_8_ bands recover. The reversible evolution of these Raman features is consistent with sulfur conversion during discharge and its partial reversal upon charging, supporting the proposed S/ZnS electrochemistry in the SHGE/TU‐based QSSZSB.

Cytocompatibility is an important consideration for gel electrolytes intended for flexible wearable and potentially bio‐integrated devices. The in vitro cytotoxicity of SHGE extracts was therefore evaluated using human dermal fibroblasts (HDF), human oral keratinocytes (HOK), and RAW 264.7 macrophages as representative cell models. HDF and HOK cells were selected to assess compatibility with cell types associated with skin and oral mucosal tissues, respectively, while RAW 264.7 macrophages were included because macrophages play important roles in inflammatory responses, wound healing, apoptotic‐cell clearance, and tissue remodeling [31, 32]. As shown in Figure 6a–c, Cell Counting Kit‐8 (CCK‐8) assays indicate that SHGE extracts produce no obvious adverse effect on the viability of HOK and HDF cells after 24, 48, and 96 h of incubation. For RAW 264.7 cells, extracts at 0.1 and 10 mg mL^−^^1^ result in partial inhibition of cell viability after 48 h, whereas after 96 h no statistically significant difference is observed relative to the control group. Overall, these results suggest favorable in vitro cytocompatibility of SHGE under the investigated exposure conditions, although some concentration‐ and time‐dependent responses are observed. Further biological evaluation would be required to establish compatibility with specific bio‐integrated applications.

**FIGURE 6:**
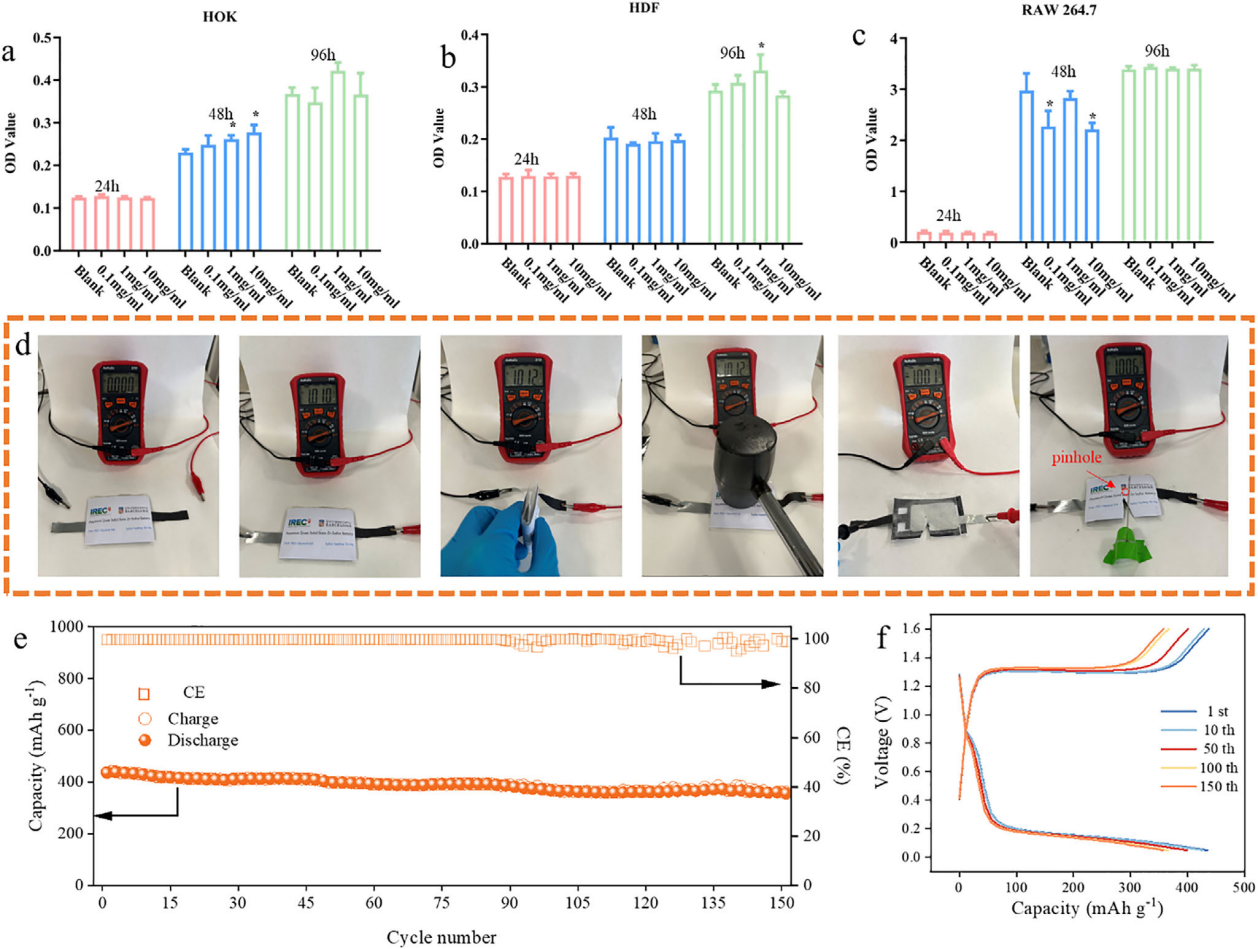
(a–c) Cell viability of (a) HOK, (b) HDF, and (c) RAW 264.7 cells after incubation with different concentrations of SHGE extract for 24, 48, and 96 h. (d) Optical photographs showing the mechanical robustness of the SHGE‐based QSS pouch cell under different conditions, including normal operation, bending, cutting, pressing, and pinhole damage. (e) Long‐term cycling performance of the SHGE‐based QSS pouch cell. (f) GCD profiles of the pouch cell at selected cycles.

The practical feasibility of SHGE in a QSS full‐cell configuration was further demonstrated using pouch cells. As shown in Figure S17, the flexible and self‐supporting SHGE can be readily bent without fracture and conformally integrated between the sulfur cathode and Zn metal anode. Such intimate electrode/electrolyte contact can facilitate continuous ion‐transport pathways while reducing the risk of electrolyte leakage associated with conventional LEs. The mechanical robustness of the assembled pouch cells was further examined under different deformation and damage conditions. As shown in Figure 6d, the SHGE‐based pouch cells retain an open‐circuit voltage of approximately 1.0 V after bending, mechanical impact, and localized damage, without visible electrolyte leakage. These observations indicate that the cells maintain their open‐circuit voltage and exhibit no apparent electrolyte leakage under the tested conditions, supporting the mechanical adaptability and electrolyte‐retention characteristics of the SHGE‐based configuration.

Long‐term cycling was further performed to evaluate the electrochemical stability of the SHGE‐based QSS pouch cells. As shown in Figure 6e, the pouch cell delivers an initial discharge capacity of approximately 435.9 mAh g^−^^1^ and retains 356.1 mAh g^−^^1^ after 150 cycles, with capacities normalized to the mass of sulfur in the cathode. The Coulombic efficiency remains above 99% throughout cycling, indicating highly reversible charge and discharge behavior. The corresponding GCD profiles (Figure 6f) retain the characteristic voltage features associated with the sulfur redox chemistry during prolonged cycling. Together, these results demonstrate that SHGE can combine Zn‐interface stabilization, mechanical adaptability, cytocompatibility, and sustained operation in a QSS full‐cell configuration, supporting its broader potential as a multifunctional electrolyte for aqueous Zn‐based batteries.

## 3 Conclusion

In summary, a glycerol‐regulated, cytocompatible SHGE was developed based on a PVA/PEG polymer matrix to stabilize Zn metal anodes in QSS aqueous batteries. Glycerol reorganizes the local hydrogen‐bonding environment and reduces polymer crystallinity, suppressing water‐related parasitic reactions while enhancing polymer‐chain mobility and ionic transport. The resulting SHGE also modifies the Zn^2^^+^ coordination environment, suppresses Zn corrosion, and promotes compact and homogeneous Zn deposition. Consequently, Zn||Zn symmetric cells sustain stable plating/stripping for more than 3300 h at 5 mA cm^−^^2^ and 5 mAh cm^−^^2^. The SHGE further exhibits favorable in vitro cytocompatibility together with good flexibility and self‐healing capability. As a proof of concept for full‐cell operation, trace TU was introduced to facilitate S/ZnS conversion, enabling sustained QSS Zn–S cycling with high Coulombic efficiency and stable pouch‐cell operation. Overall, this work demonstrates a multifunctional gel‐electrolyte strategy that combines Zn‐interface stabilization, mechanical adaptability, and cytocompatibility for durable QSS aqueous Zn‐based batteries.

## Author Contributions

**Yuchuan Ren**: conceptualization, methodology, writing – original draft, writing – review and editing, data curation, validation, visualization. **Qian Xue**: conceptualization, software. **Lihong He**: data curation. **Canhuang Li**: writing – original draft, conceptualization, data curation. **Ruixia Li**: data curation. **Xiaoyu Bi**: conceptualization, visualization. **Jiali Chai**: software, data curation. **Yapeng Cheng**: data curation, visualization. **Tiezhu Guo**: writing – review and editing, writing – original draft, conceptualization, supervision. **Andreu Cabot**: conceptualization, writing – review and editing, supervision, resources, funding acquisition, project administration.

## Conflicts of Interest

The authors declare no conflicts of interest.

## Supporting information




**Supporting File**: advs78625‐sup‐0001‐SuppMat.docx.

## Data Availability

Data are available from the corresponding authors upon reasonable request.
