# Supplementary material for: Glycerol‐Regulated Cytocompatible, Self‐Healing Gel Electrolyte Stabilizes Zinc Metal Anodes for Quasi‐Solid‐State Aqueous Batteries
Source: Adv Sci (Weinh). 2026 Nov 3:e78625. Online ahead of print. doi: 10.1002/advs.78625 (PMC13731083; doi:10.1002/advs.78625)
Supplement: Supplementary file 1 [file ADVS-9999-e78625-s001.docx]

**Glycerol-Regulated Cytocompatible, Self-Healing Gel Electrolyte Stabilizes Zinc Metal Anodes for Quasi-Solid-State Aqueous Batteries**

Yuchuan Ren^a, b^, Qian Xue^a, b^, Lihong He^c, d, e^, Canhuang Li^f^*, Ruixia Li^g^, Xiaoyu Bi^a, b^, Jiali Chai^a, b^, Yapeng Cheng^a, b^, Tiezhu Guo^a, h^*, Andreu Cabot^a, i^*

a.	Catalonia Energy Research Institute – IREC, Sant Adrià de Besòs 08930, Barcelona, Catalonia, Spain.

b.	Departament d’Enginyeria Electrònica i Biomèdica, Universitat de Barcelona, 08028, Barcelona, Catalonia, Spain.

c.	Hospital of Stomatology, Sun Yat-sen University, Guangzhou, China.

d.	Guangdong Provincial Key Laboratory of Stomatology, Guangzhou, China.

e.	Guanghua School of Stomatology, Sun Yat-sen University, Guangzhou, China.

f.	State Key Laboratory of Tropic Ocean Engineering Materials and Materials Evaluation, School of Marine Technology and Equipment, Hainan University, 570228, Haikou, China.

g.	State Key Laboratory of Cancer Biology, National Clinical Research Center for Digestive	Diseases and Xijing Hospital of Digestive Diseases, 710032, Xi'an, China

h.	Multifunctional Materials and Structures, Key Laboratory of the Ministry of Education & International Center for Dielectric Research, School of Electronic Science and Engineering, Xi’an Jiaotong University, 710049, Xi’an, PR China.

i.	ICREA, Pg. Lluis Companys 23, Barcelona, 08010, Catalonia, Spain.

**Experimental section**

**1. Chemicals**

All chemicals were used as received without further purification. Polyvinyl alcohol 6000 (PVA), polyethylene glycol 13000 (PEG), sulfur, zinc trifluoromethanesulfonate, thiourea, and glycerol were bought from Sigma-Aldrich. Guar gum, zinc foil, hydrophilic carbon cloth, and glass fiber separator were purchased from Canrud Company, China.

**2. Preparation of Electrolytes**

First, 75.46 g of Zn(OTF)_2_ was dissolved in 100 mL of deionized water to prepare a 2 M Zn(OTF)_2_ solution, which was used as the liquid electrolyte (LE). To prepare SHGE, PVA and PEG were separately dissolved in deionized water at a concentration of 15 wt% under magnetic stirring at 80 °C for 12 h. The resulting PVA and PEG solutions were then mixed at a PVA:PEG mass ratio of 3:1. The homogeneous mixture was poured into a polytetrafluoroethylene (PTFE) mold and dried at 50 °C to remove water and form a PVA/PEG film. The dried film was subsequently immersed in 2 M Zn(OTF)_2_ solution for 12 h. The resulting PVA/PEG/Zn(OTF)_2_ gel was then immersed in a minimal amount of glycerol sufficient to fully cover the gel and left for 12 h to allow glycerol incorporation into the polymer network. Afterward, the sample was left at room temperature until the excess liquid surrounding the gel had evaporated, yielding the final SHGE. Based on the formulation composition, the final glycerol content was approximately 10–15 wt%, while the water content was approximately 50 wt%. The SHGE used for electrochemical measurements had a thickness of approximately 200 μm.

For the SHGE used in Zn–S battery tests, thiourea was added to the 2 M Zn(OTF)_2_ soaking solution at 0.05 wt%. Thiourea was therefore incorporated during the soaking step and retained in the gel during subsequent preparation.

**3. Sulfur Cathode Preparation**

Super P and sulfur powder were mixed at a mass ratio of 1:1, thoroughly ground, and heated at 155 °C for 12 h to obtain the S/C composite. The S/C composite and guar gum binder were then mixed at a mass ratio of 9:1 and dispersed in deionized water under stirring for 12 h to obtain a homogeneous slurry. The slurry was coated onto hydrophilic carbon cloth and dried in a vacuum oven at 60 °C for 12 h. The resulting electrodes were punched into 10 mm diameter disks for cell assembly.

**4. Electrochemical test**

Ionic conductivity of LE, PVA/PEG, and SHGE was measured using a Luton CD-4039 portable handheld conductivity meter (the error is a relative error calculated based on the average of measurements). The gel was prepared as a sufficiently large bulk sample to allow complete insertion of the probe into the interior of the gel. Before each measurement, the conductivity meter was calibrated using a standard conductivity solution. The probe was fully embedded in the gel to ensure intimate contact between the sensing electrodes and the hydrated polymer network. Measurements were conducted at room temperature, and at least three measurements were collected at different positions of each sample. The average value was reported as the conductivity of the hydrated gel electrolyte [1]. Electrochemical measurements were performed using CR2032-type coin cells with glass-fiber separators and 100-μm-thick Zn foil. Linear sweep voltammetry (LSV) was performed using a CS2350 electrochemical workstation (Corrtest, China) to evaluate the electrochemical stability windows of LE, the PVA/PEG gel electrolyte, and SHGE. A platinum mesh was used as the working electrode, a glassy carbon electrode as the counter electrode, and an Ag/AgCl electrode as the reference electrode[2]. Zn nucleation behavior was evaluated by cyclic voltammetry using Zn//Cu cells with a BCS-805 electrochemical workstation (BioLogic, USA). Chronoamperometry measurements were performed using a CS2350 electrochemical workstation in Zn//Zn symmetric cells at a fixed overpotential of −150 mV to investigate Zn deposition behavior. To examine the morphology of deposited Zn, Zn was plated onto Cu substrates at areal capacities of 1, 5, and 10 mAh cm^-2^ at a current density of 5 mA cm^-2^, followed by SEM characterization. Long-term cycling of Zn//Zn symmetric cells and galvanostatic charge/discharge measurements of Zn–S cells were performed using a computer-controlled multichannel battery testing system (Neware, China). The Coulombic efficiency (CE) of Zn plating/stripping was evaluated using Zn//Cu asymmetric cells at a current density of 2 mA cm⁻² and an areal plating capacity of 1 mAh cm⁻². The CE was calculated as the ratio between the stripped and plated capacities during each cycle.

**5. Materials Characterization**

Fourier-transform infrared spectroscopy (FTIR) was performed by Alpha-p (Bruker) to analyze the intermolecular interactions within the gel electrolytes. X-ray diffraction (XRD, Bruker D8 ADVANCE) was used to investigate the crystalline structure of the polymer electrolytes and the corrosion products on Zn foils after immersion. Scanning electron microscopy (SEM, Ultra-55, Zeiss, Germany) and a confocal optical microscope were used to observe the morphology of Zn foils after immersion, deposited Zn at different areal capacities, and Zn electrodes after cycling. Optical photographs were taken to demonstrate the flexibility, self-healing behavior, pouch-cell assembly process, and safety characteristics of the SHGE-based quasi-solid-state cells. Raman spectroscopy and in situ Raman measurements were carried out to monitor the sulfur conversion process during discharge and charge. The gel water loss rate is determined by placing the gel in a Petri dish, sealing it with plastic wrap, allowing it to stand for a specific period of time, and then calculating the weight loss.

**6. Theoretical method**

Molecular dynamics simulations were performed using the Materials Studio software package; both poly(vinyl alcohol) (PVA) and poly(ethylene glycol) (PEG) were represented by polymer chains with a degree of polymerization of 20. In contrast, a PVA oligomer containing three repeating units was employed for the desolvation-energy calculations. The optimized PVA trimer was used to evaluate the replacement of coordinated water molecules by PVA hydroxyl groups around Zn^2+^.

The initially constructed configurations were first subjected to geometry optimization using the Forcite module to eliminate unfavorable atomic overlaps and high-energy contacts. All interatomic interactions were described using the COMPASS II force field. The convergence criteria for energy, force, and atomic displacement were set to 1x10-4 kcal mol-1, 0.005 kcal mol^-1^ Å^-1^, and 5x10-5 Å, respectively. Van der Waals interactions were calculated using the atom-based method.

After geometry optimization, molecular dynamics simulations were performed under the NVT ensemble to equilibrate the electrolyte systems and collect trajectories for structural analysis. The simulation temperature was maintained at 298 K, and the time step was set to 1.0 fs. Each system was equilibrated for 500 ps. Identical simulation procedures and parameters were applied to all systems to ensure reliable comparison among different electrolyte compositions.

In particular, desolvation-energy calculations were performed based on the optimized coordination structures obtained using the Forcite module in Materials Studio with the COMPASS II force field. All isolated species and Zn-containing complexes were fully optimized before energy evaluation.

For the hydrated Zn^2+^ reference system, the initial coordination structure was constructed as Zn-(H₂O)₆. The six coordinated water molecules were sequentially removed. The dehydration energy for each sequential water-removal step was calculated as:

$$\text{Δ}\text{E}_{\text{H}_{\text{2}}\text{O,i}}\text{ = }\text{E}_{\text{Zn-}\left( \text{H}_{\text{2}}\text{O} \right)_{\text{6-i}}}\text{ + }\text{E}_{\text{H}_{\text{2}}\text{O}} \text{-}\text{ E}_{\text{Zn-}\left( \text{H}_{\text{2}}\text{O} \right)_{\text{7-i}}}\text{ }\left( \text{i = 1,2,3,4,5,6} \right)$$

where i denotes the water-removal step, $\text{E}_{\text{Zn-}\left( \text{H}_{\text{2}}\text{O} \right)_{\text{7-i}}}$ and $\text{E}_{\text{Zn-}\left( \text{H}_{\text{2}}\text{O} \right)_{\text{6-i}}}$ represent the total energies of the Zn^2+^ coordination structures before and after the removal of one coordinated water molecule, respectively. $\text{E}_{\text{H}_{\text{2}}\text{O}}$ is the energy of an isolated water molecule.

For the PVA/OTF-containing coordination structure, the initial complex was constructed as $\text{Zn-PVA-OTF-}\left( \text{H}_{\text{2}}\text{O} \right)_{\text{4}}$ complex, where Zn^2+^ was coordinated with PVA, one OTF- anion, and four water molecules. The four coordinated water molecules were first removed stepwise. The dehydration energy for each sequential water-removal step was defined as:

$$\text{Δ}\text{E}_{\text{H}_{\text{2}}\text{O,i}}\text{ = }\text{E}_{\text{Zn-PVA-OTF-}\left( \text{H}_{\text{2}}\text{O} \right)_{\text{4-i}}}\text{ + }\text{E}_{\text{H}_{\text{2}}\text{O}} \text{- }\text{E}_{\text{Zn-PVA-OTF-}\left( \text{H}_{\text{2}}\text{O} \right)_{\text{5-i}}}\text{ }\left( \text{i = 1,2,3,4} \right)$$

where i denotes the water-removal step, $\text{E}_{\text{Zn-PVA-OTF-}\left( \text{H}_{\text{2}}\text{O} \right)_{\text{5-i}}}$ and $\text{E}_{\text{Zn-PVA-OTF-}\left( \text{H}_{\text{2}}\text{O} \right)_{\text{4-i}}}$ are the total energies of the coordination complexes before and after the removal of one coordinated water molecule, respectively. After complete removal of the four coordinated water molecules, the detachment energies of PVA and OTF^-^ were calculated as:

$$\text{Δ}\text{E}_{\text{PVA}}\text{ =}\text{ E}_{\text{Zn-OTF}}\text{ + }\text{E}_{\text{PVA}} \text{-}\text{ E}_{\text{Zn-PVA-OTF}}$$

$$\text{Δ}\text{E}_{\text{OTF}}\text{ =}\text{ E}_{\text{Zn}}\text{ + }\text{E}_{\text{OTF}} \text{- }\text{E}_{\text{Zn-OTF}}$$

where $\text{E}_{\text{Zn-PVA-OTF}}$ is the energy of the fully dehydrated Zn-PVA-OTF complex, $\text{E}_{\text{Zn-OTF}}$ is the energy of the Zn-OTF complex after PVA removal, $\text{E}_{\text{Zn}}$ is the energy of the isolated Zn^2+^ species, $\text{E}_{\text{PVA}}$ is the energy of the isolated PVA oligomer model, and $\text{E}_{\text{OTF}}$ is the energy of the isolated OTF^-^ anion. In all cases, a larger positive ΔE indicates a higher energy requirement for removing the corresponding coordinated species from the Zn^2+^ solvation structure.

**7. Cytotoxicity evaluation**

The SHGE material was soaked in 75% alcohol for 30 min, rinsed with ultrapure water, and then soaked in DMEM supplemented with 10% fetal bovine serum (FBS) and 100 μg mL^-1^ streptomycin/penicillin mixture at the ratio of 0.1 mg mL^-1^, 1 mg mL^-1^, and 10 mg mL^-1^, respectively. The SHGE was soaked at 37°C for 24 hours to obtain extracts, and then the cell culture medium was filtered with a 0.22  μm filter. In subsequent experiments, the Cell Counting Kit-8 (CCK-8) was used to estimate the number of cells to assess cell viability. Human dermal fibroblasts (HDF), human oral keratinocytes (HOK), and macrophage cell line RAW 264.7 were seeded in 96-well plates with a density of 1 × 10^3^ cells/well and cultured at 37 °C with a 5% CO_2_ atmosphere. After receiving the corresponding electrolyte leaching solution at different concentrations, the cell proliferation ability was detected by CCK-8 reagent at 24, 48, and 96 h according to the manufacturer’s instructions. Briefly, 10 μl CCK-8 solution was dripped into each well and incubated at 37℃ in the dark for 4 h, and the optical density (OD values) was measured at 450 nm using a microplate reader. The relative cell viability of cells was analyzed according to OD values.

Statistical analysis: The results of CCK8 assays are presented as mean ± standard deviation (SD) and analyzed using an unpaired t-test. In all cases, P < 0.05 was considered statistically significant. * Denotes *P* < 0.05.

**Figure S1.** Photographs of the gel electrolyte wrapped around a straight tube and the self-supporting, self-healing structure.

**Figure S2.** Weight change of SHGE over 15 days at room temperature.

**Figure S3.** FTIR curves of PVA/PEG gel and SHGE.

**Figure S4.** XRD patterns of PVA/PEG gel and SHGE.

**Figure S5.** Measured ionic conductivity of LE, PVA/PEG, and SHGE.

**Figure S6.** Ionic conductivity of pristine SHGE and after the first and second healing cycles.

**Figure S7.** a) Radial distribution function and coordination number of Zn–O(H₂O), b) Simulated electrolyte configuration. c) Representative local coordination structure of Zn²⁺.

**Figure S8.** Radial distribution functions and corresponding coordination numbers of a) Zn–O(OTF), b) Zn–O(PVA), and c) Zn–O(PEG) in the PVA/PEG gel electrolyte.

**Figure S9.** Radial distribution functions and corresponding coordination numbers of a) Zn–O(OTF), b) Zn–O(PVA), c) Zn–O(PEG), and d) Zn–O(glycerol) in SHGE.

**Figure S10.** Optimized molecular configurations of the stepwise desolvation process for hydrated Zn^2+^ in the LE.

**Figure S11.** Optimized molecular configurations of the stepwise desolvation process for Zn²⁺ in the SHGE system.

**Figure S12**. a) Galvanostatic Zn nucleation profiles of Zn||Cu cells using LE and SHGE, and corresponding Zn deposition morphologies on Cu at an areal capacity of 0.5 mAh cm⁻² based on SHGE (b) and LE (c)

**Figure S13.** Coulombic efficiency of Zn//Cu asymmetric cells based on LE and PVA/PEG.

**Figure S14.** Long-term galvanostatic cycling performance of Zn||Zn symmetric cells using PVA/PEG gel electrolyte at 5 mA cm⁻² and 5 mAh cm⁻².

**Figure S15.** Thermogravimetric curves of the sulfur/carbon composite conducted under an N_2_ atmosphere.

**Figure S16.** Cycling performance of a Zn–S cell using the glycerol-free PVA/PEG gel electrolyte containing 0.05 wt% thiourea at 1 A g⁻¹.

**Figure S17.** a, b) Optical photographs of the SHGE, sulfur cathode, and zinc anode assembly; c) Optical photograph of the sulfur cathode//SHGE//Zn anode in a bent configuration.

Reference

1. W. Guo, F. Gu, Q. Chen, et al., “Photothermal-boosted flexible rechargeable zinc-air battery based on Ni-doped Mn_3_O_4_ with excellent low-temperature adaptability” *Carbon Energy* 6, no.11 (2024): e567, https://doi.org/10.1002/cey2.567

2. Y. Qian, L. Su, H. Jing, et al., “Extended Electrochemical Window Via Interfacial Microdomain Regulation by a Bicontinuous Microemulsion-Based Heterogel Electrolyte” *Carbon Energy* 7, no.5 (2025): e697, https://doi.org/10.1002/cey2.697
